# Association between knowledge of Human Immunodeficiency Virus transmission and consistent condom use among sexually active men in Nigeria: An analysis of 2018 Nigeria Demographic Health Survey

**DOI:** 10.1371/journal.pgph.0000223

**Published:** 2022-03-21

**Authors:** Obasanjo Afolabi Bolarinwa, Kobi V. Ajayi, Rajeeb Kumar Sah

**Affiliations:** 1 Department of Global Public Health, School of Allied and Public Health Professions, Canterbury Christ Church University, Canterbury, United Kingdom; 2 Department of Public Health Medicine, School of Nursing and Public Health, University of KwaZulu-Natal, Durban, South Africa; 3 Obaxlove Consult, Lagos, Nigeria; 4 Education, Direction, Empowerment, & Nurturing (EDEN) Foundation, Abuja, Nigeria; 5 Department of Health and Kinesiology, Texas A&M University, College Station, TX, United States of America; 6 Laboratory for Community Health Evaluation and Systems Science (CHESS), Texas A&M University, College Station, TX, United States of America; 7 School of Human and Health Sciences, University of Huddersfield, Queensgate, Huddersfield, United Kingdom; Wollega University, ETHIOPIA

## Abstract

An estimated 1.7 million people were living with HIV in Nigeria in 2020, with over 86,000 people newly infected. Although the global rates of HIV have remained consistent over time, Nigeria has the second-highest number of people living with HIV and contributes to 9% of the global burden of HIV/AIDS. This is due to several structural and individual-level factors that limit knowledge of HIV and condom utilization. In this context, this study examines the association between knowledge about HIV transmission and consistent condom use among sexually active men in Nigeria. The data utilised in this study was sourced from the latest Nigeria Demographic and Health Survey conducted in 2018. The sample included a total of 9,346 men between the ages of 15–59 years who were sexually active at the time of data collection. Frequency distribution, univariate and multivariable analyses were performed at 95% confidence interval and p-value less than 0.05 to determine the association between the key independent variables and covariates. The results showed that 85.03% of sexually active men who had no knowledge of HIV engaged in inconsistent condom use. The key independent variable showed that sexually active men who had knowledge of HIV had higher odds [AOR = 1.37; 95%(CI = 1.10–1.72)] of consistent condom use compared to those without knowledge of HIV. However, sexually active men who were previously married [AOR = 0.38; 95%(CI = 0.24–0.61)], and those residing in the South Eastern region of Nigeria [AOR = 0.62; 95%(CI = 0.44–0.96)] had lower odds of consistent condom use. This study established the association between HIV knowledge and consistent condom use among sexually active males in Nigeria even after controlling for confounders. Educational level, wealth index, and ethnicity are also associated with condom use. This calls for the consideration of social determinants of health, localised and cultural health promotion and targeted public health strategies at all governmental levels to combat the HIV/AIDS epidemic in Nigeria.

## Background

As of 2020, an estimated 1.7 million people were living with HIV in Nigeria, with over 86,000 newly infected in that year [1]. Although the total number of people living with HIV globally has remained consistent over time (i.e., less than 25% change upwards or downwards), Nigeria has the second-highest number of people living with HIV and contributes to 9% of the global burden of HIV/AIDS [2,3]. Thus, understanding the underlying factors contributing to the unacceptably high HIV burden in the country is pertinent.

Empirical evidence suggests that increased HIV-related knowledge is associated with a reduced risk for HIV infection and transmission. Report from the Nigerian Demographic and Health Surveys (NDHS) indicate that between 2003 to 2013, there was a minimal mean increase in general HIV-related knowledge and HIV-risk reduction among the general population [4]. Despite the increase in HIV knowledge, the prevalence of HIV in Nigeria is still higher than its neighboring West and Central African countries combined, with a prevalence of 3.4% compared to 2.2% [5]. Yet, in 2012 only 25% of the general population in Nigeria reported having comprehensive knowledge of HIV transmission and prevention. Sadly, this rate has remained stable with only 25.4% of adults and 24.4% of young people aged 15–24 in Nigeria, with comprehensive knowledge about HIV transmission as of the end of 2015 [6]. This is despite the years of increase in international efforts to address the HIV epidemic in the country. Through the President’s Emergency Plan for AIDS Relief (PEPFAR) provides HIV therapy to upwards of 90% of people living with HIV/AIDS in the country [2,7]. Given these indicators, improving awareness and knowledge of HIV-related preventive behaviour is fundamental towards HIV reduction and transmission.

Other than abstinence, consistent and correct condom use is the most effective mechanism of preventing the transmission of HIV, particularly among young adults. Moreover, condoms are effective in preventing higher-risk sexual behaviours (e.g., anal or vaginal coitus) and lower-risk sexual activities (e.g., oral sexual activities) [8]. With the AIDS epidemic, there has been no shortage of calls to increase safer sex practices through the use of condoms to inform public health interventions, programs, and policy. Moreover, consistent condom use has been heralded as fundamental to reducing unwanted pregnancies and abortion rates. However, despite its potency, there is an inconsistent or underutilisation of condoms among sexually active people in sub-Saharan Africa as in Nigeria [9,10]. A survey conducted by the national think tank in Nigeria revealed that while 92% of Nigerians know about condoms, only 34% of Nigerians use condoms [11]. This low rate coincides with national estimates where only 17% of young, never-married males used condoms at their sexual debut [12]. However, slightly above average at 55% of the general population (male and female) reported consistently using condoms with a non-marital sex partner [6]. Considering the association between condom use and HIV transmission, creating strategies to increase HIV knowledge and consistent condom use in Nigeria is a public health priority.

The reasons young adults do not effectively and consistently use condoms are well documented. Studies in Nigeria and elsewhere show that age, educational level, type and number of partners, religious affiliation, place of residence, alcohol use, exposure to media, perceived sexual dissatisfaction from condom use, low perception about risky sexual behaviours and STDs are some barriers to consistent condom use [13]. However, inherent societal attitudes and beliefs in Nigeria also moderate consistent condom use. For example, there are insurmountable social pressures among married people to procreate, and because of this, condoms are rarely used, whether for STDs or as a contraceptive. As a result, there is a considerable high rate of inconsistent condom use and discontinuation of condoms by married couples in Nigeria. Males also hold the stereotypical belief that condom use is associated with negative health outcomes in women, including inflammation and diseases [14,15]. Conversely, males and females also perceive condom use as harmful or divulge from God’s original intent of their reproductive organs [15].

Although males tend to use condoms more than females in Nigeria overall, males constitute a key population that accounts for a disproportionate share of the HIV burden [6]. Between 2007 and 2014, the HIV prevalence among men who have sex with men (MSM) increased from 13.5% to 22.9%. In addition, the HIV prevalence among this population is 3 to 10 times higher than the general population [16]. Yet, due to the stigmatisation and criminalisation of homosexuality in the country, most MSM practice bisexuality and despite their HIV status, have unprotected heterosexual relationships with married and unmarried females [16]. Males in Nigeria, as in other patriarchal societies, play an integral role in the acceptance and utilisation of contraceptives, including condoms by their partners [17]. MSMs, compared to the general population, do not perceive themselves at an increased risk of HIV infection, suggesting a low HIV-related knowledge [6,16] and therefore contributing to the higher burden of HIV prevalence among females and the country as a whole.

Against this backdrop, it is imperative to investigate knowledge about HIV transmission and its implication on consistent condom use among males to improve both males’ and females’ sexual and reproductive health outcomes and inform public health practice and policy. Although prior studies in Nigeria have attempted to understand this phenomenon, they are limited to reproductive-aged women, adolescents, or other HIV-related constructs [4]. Therefore, the purpose of this study is to examine whether HIV transmission-related knowledge moderates consistent condom use among sexually active men in Nigeria. In keeping with previous studies, we hypothesize that poor HIV knowledge will be associated with inconsistent condom use.

## Methods

### Design and setting of the study

We used data from the most recent cross-sectional Nigerian Demographic Health Survey (NDHS) released in 2018 to examine the association between HIV-related knowledge and consistent condom use among sexually active men in Nigeria. This is a nationally representative dataset conducted in more than eighty-five (85) low-and-middle-income countries (LMICs). The NDHS 2018 data collection was conducted between the 14th of August to 29th of December 2018 using a questionnaire from respondents on several health indicators relating to sexual and reproductive health, including condom use, HIV knowledge, gender-based violence, fertility intention and family planning use [18,19]. As detailed by Aliaga and Ruilin [20], the DHS sampling process of the respondents involved the use of a two-stage cluster random sampling technique.

A total of 41,668 households were selected for the sample, of which 40,666 were occupied. Of the occupied households, 40,427 were successfully interviewed, yielding a response rate of 99%. In the subsample of households selected for the male survey, 13,422 men aged 15–59 were identified, and 13,311 were successfully interviewed, yielding a response rate of 99%. However, the respondents that met this study criteria inclusion were 9,346 sexually active men. The NDHS survey captures data on modern contraceptive usage and HIV knowledge. The survey also captures respondents’ socio-demographic characteristics, which can be used to control the association between HIV-related knowledge and consistent condom among the target population [19].

### Ethics statement

Since the authors of this manuscript did not collect the data, we sought permission from the MEASURE DHS website, and access to the data was provided after our intent for the request was assessed and approved on the 10th of January 2021, and a de-identified dataset was downloaded from DHS website. The DHS surveys are ethically accepted by the ORC Macro Inc. Ethics Committee and the Ethics Boards of partner organizations in different countries, such as the Ministries of Health. The men interviewed in this survey gave either written or verbal consent for participation.

### Dependent variable

The dependent variable in this study was consistent condom use among sexually active men in Nigeria. Men who were sexually active in the last 4 weeks were categorised as sexually active [21]. During the DHS surveys’ interviews, men were asked if “condom used during last sex with a most recent partner?”, or “condom used during last sex with 2nd to most recent partner?”, or “condom used during last sex with 3rd to most recent partner?” those who answered “yes” were categorised as consistent condom use during sexual intercourse while those who said “no” were categorised as inconsistent condom use during sexual intercourse. The survey responses in this study were dichotomised as 0 = “inconsistent condom use” and 1 = “consistent condom use”. This study’s categorisation and variable selections align with previous related studies [12,22–24].

### Key independent variable

In this study, the key independent variable was knowledge of HIV transmission, and this was selected based on its direct influence on adverse sexual and reproductive health outcomes [2,25]. Respondents were asked whether HIV can be transmitted from mother to her baby ‘during pregnancy’, ‘during delivery’, ‘by breastfeeding’. The questions, “Does HIV transmit during sex?", “Does HIV transmit during delivery?" and “Does HIV transmit by breastfeeding?" Respondents that answered “no” to either of the three questions were coded as “0,” representing “had no knowledge of HIV,” while those who responded “yes” to the three questions were coded as “1” representing “had knowledge of HIV” [26,27].

### Covariates

The covariates selected in this study were based on previous similar studies on condom use globally and the availability of the variables in the NDHS dataset [23,24]. The covariates constitute the second explanatory domain and are included in the analysis to control for possible confounders in the study regression analytical model. Socio-demographic variables included were age group, level of education, marital status, working status, ethnicity, religion, mass media, wealth index, place of residence, and regions. A composite variable was used to generate the mass media variable through the three survey questions that were asked about the respondent’s exposure to mass media, which were frequencies of watching television, listening to the radio, and reading newspapers or magazines. The additive scale of the composite variable generated for media was grouped into a binary-level category such as 1 = “Yes, for respondent with atleast one of the mass media exposure” and 0 = “No, for respondents with no exposure to any of the three mass media.” In the NDHS, Principal Component Analysis (PCA) was used to generate wealth index from a range of information collected on household assets, including type and volume of household features, consumer goods owned, ownership of animals, and means of transportation [28].

### Data analyses

The distribution of sexually active men in Nigeria was first described across HIV transmission knowledge, the selected covariates and consistent condom use. This was followed by examining the associations between consistent condom use, key independent variables, and other explanatory variables using the chi-square test. Both univariate and multivariable logistic regression models were later set up to examine both unadjusted odds ratio (cOR) and adjusted odds ratios (AOR) effects of respondent’s HIV knowledge and selected covariates on consistent condom use at 95% confidence intervals (CIs). In the NDHS dataset, there is a possibility of variations in response rates across different states because the survey samples were not proportionally allocated to the sub-nationals to account for the complex survey sampling design and ensure representativeness of the model estimates at the national level, all frequency distribution analyses we weighted using the “SVY” stata command. No multicollinearity was observed among the explanatory variables. All data were analyzed using STATA, version 16.0.

## Results

The results showed that 85.30% of sexually active men who had no knowledge of HIV transmission engaged in inconsistent condom use while 14.70% engaged in consistent condom use. In the covariates, 89.21% of respondents aged 35 and above did not engage in consistent condom use. 96.91% of sexually active men with no educational background reported inconsistent condom use. Both key independent variables and selected covariates were associated with consistent condom use (Table 1).

**Table 1:** Weighted distribution and chi-square analysis of sexually active men by consistent condom use in Nigeria (N = 9,346).

Variables	Frequency	Percentage	Consistent condom use (%)		
Key independent variable			No	Yes	p-Value (χ^2^)
**Knowledge of HIV transmission**					p<0.01
Had no knowledge	1,746	18.68	85.30	14.70	
Had Knowledge	7,600	81.32	81.50	18.50	
**Covariates**					
**Age of respondents**					p<0.001
15–24	810	8.67	49.91	50.09	
25–34	2,795	29.91	77.18	22.82	
35 & above	5,741	61.42	89.21	10.79	
**Educational level**					p<0.001
No education	2,157	23.07	96.91	3.09	
Primary	1,471	15.74	89.75	10.25	
Secondary	3,902	41.75	74.67	25.33	
Higher	1,816	19.43	74.83	25.17	
**Marital Status**					p<0.001
Not married	1,267	13.56	31.97	68.03	
Currently married	7,639	81.73	90.23	9.77	
Cohabiting	352	3.77	93.04	6.96	
Previously married	89	0.95	65.70	34.30	
**Working status**					p<0.001
No	426	4.56	46.48	53.52	
Yes	8,920	95.44	83.91	16.09	
**Ethnicity**					p<0.001
Hausa	2,920	31.25	95.89	4.11	
Yoruba	1,744	18.66	75.77	24.23	
Igbo	1,522	16.29	75.04	24.96	
Others	3,160	33.81	76.56	23.44	
**Religious**					p<0.001
Christianity	4,657	49.83	72.82	27.18	
Islam	4,608	49.30	91.68	8.32	
Traditionalist & Others	81	0.87	83.24	16.76	
**Mass media exposure**					p<0.001
Not exposed	2,002	21.42	91.25	8.75	
Exposed	7,344	78.58	79.74	20.26	
**Wealth index**					p<0.001
Poorest	1,436	15.37	95.68	4.32	
Poorer	1,586	16.97	89.91	10.09	
Middle	1,847	19.76	82.88	17.12	
Richer	2,081	22.26	76.21	23.79	
Richest	2,396	25.63	73.72	26.28	
**Place of residence**					p<0.001
Urban	4,431	47.41	77.63	22.37	
Rural	4,915	52.59	86.33	13.67	
**Region of residence**					p<0.001
North Central	1,293	13.84	81.03	18.97	
North East	1,382	14.78	91.33	8.66	
North West	2,178	23.30	94.60	5.40	
South East	1,168	12.50	76.69	23.31	
South South	1,282	13.72	64.11	35.89	
South West	2,043	21.86	78.09	21.91	
Total	9,346	100.00	82.21	17.79	

Weighted NDHS 2018.

The adjusted (model II) in Table 2 showed a significant association between age at sexual debut and consistent condom use among sexually active men in Nigeria. Among the selected covariates, the significant variables included educational level, marital status, ethnicity, religion, wealth index, and region of residence.

**Table 2:** Bivariate and multivariable logistics regression analysis of knowledge of HIV and consistent condom use among sexually active men in Nigeria (N = 9,346).

Variables	Unadjusted (Model I)	Adjusted (Model II)
	cOR [95% CI]	aOR [95% CI]
**Key independent variable**		
**Knowledge of HIV**		
Had no knowledge	1	1
Had knowledge	1.32**[1.10–1.58]	1.37**[1.10–1.72]
**Covariates**		
**Age of respondents**		
15–24	1	1
25–34	0.29***[0.23–0.37]	1.02[0.76–1.36]
35 & above	0.12***[0.10–0.15]	0.86[0.63–1.17]
**Educational level**		
No education	1	1
Primary	3.58***[2.40–5.33]	1.60*[1.01–2.53]
Secondary	10.63***[7.57–14.91]	2.36***[1.56–3.57]
Higher	10.54***[7.34–15.13]	2.32***[1.46–3.68]
**Marital Status**		
Not married	1	1
Currently married	0.05***[0.04–0.06]	0.08***[0.06–0.10]
Cohabiting	0.03***[0.02–0.06]	0.03***[0.02–0.05]
Previously married	0.24***[0.16–0.38]	0.38***[0.24–0.61]
**Working status**		
No	1	1
Yes	0.17***[0.12–0.22]	0.94[0.63–1.41]
Ethnicity		
Hausa	1	1
Yoruba	7.45***[5.62–9.88]	3.42***[1.97–5.96]
Igbo	7.75***[5.88–10.22]	2.39**[1.36–4.21]
Others	7.13***[5.48–9.28]	1.90*[1.15–3.16]
Religious		
Christianity	1	1
Islam	0.24***[0.21–0.28]	0.57***[0.44–0.73]
Traditionalist & Others	0.54[0.26–1.11]	1.05[0.49–2.28]
Mass media exposure		
Not exposed	1	1
Exposed	2.65***[2.17–3.24]	1.21[0.94–1.56]
Wealth index		
Poorest	1	1
Poorer	2.48***[1.71–3.60]	1.34[0.88–2.03]
Middle	4.57***[3.27–6.40]	1.56*[1.02–2.38]
Richer	6.91***[4.97–9.61]	1.80**[1.18–2.76]
Richest	7.89***[5.66–11.01]	1.69*[1.08–2.64]
Place of residence		
Urban	1	1
Rural	0.55***[0.48–0.63]	0.86[0.72–1.04]
Region of residence		
North Central	1	1
North East	0.40***[0.30–0.54]	0.76[0.55–1.06]
North West	0.24***[0.17–0.35]	0.84[0.51–1.37]
South East	1.30*[1.04–1.62]	0.58**[0.40–0.84]
South South	2.39***[1.95–2.92]	1.20[0.92–1.57]
South West	1.20[0.97–1.48]	0.65*[0.44–0.96]

Weighted NDHS, 2018.

RC = Recode; CI = confidence interval; cOR = unadjusted odds ratios; aOR = adjusted odds ratios

* p < 0.05

** p< 0.01

*** p < 0.001.

The key independent variable showed that sexually active men who had knowledge of HIV had higher odds [AOR = 1.37; 95%(CI = 1.10–1.72)] of consistent condom use compared to those without knowledge of HIV.

Among the selected covariates, the results show that sexually active men who had secondary education [AOR = 2.36; 95%(CI = 1.56–3.57)], higher education [AOR = 2.32; 95%(CI = 1.46–3.68)], those whose ethnicity was Yoruba [AOR = 3.42; 95%(CI = 1.97–5.96)], Igbo [AOR = 2.39; 95%(CI = 1.36–4.21)], and sexually active men from richest wealth index [AOR = 1.69; 95%(CI = 1.08–2.64)] had higher odds of engaging in consistent condom use during sexual intercourse compared to sexually active men who were not educated, those from Hausa ethnic group, and sexually active men from poorest wealth index while sexually active men who were previously married [AOR = 0.38; 95%(CI = 0.24–0.61)], those practicing Islam [AOR = 1.69; 95%(CI = 0.44–0.73)], and sexually active men residing in South Eastern region of Nigeria [AOR = 0.62; 95%(CI = 0.44–0.96)] had lower odd of consistent condom use compared to sexually active women who were not married, those practicing Christianity, and those residing in North Central.

## Discussion

This study aimed to assess the association between HIV transmission knowledge and consistent condom use among sexually active men in Nigeria. After adjusting for the confounders, the study showed that sexually active men in Nigeria with HIV knowledge had higher odds of consistent condom use than men without HIV knowledge. Our findings are similar to those found in Nigeria, Sub-Saharan Africa, and elsewhere showing that exposure to HIV-related information promotes consistent and efficient condom use [24,29,30]. This finding demonstrates the importance of providing strategies that improve HIV awareness and prevention efforts for safe sex practices. Moreover, these strategies could dispel negative beliefs while also destigmatizing HIV/AIDS, thereby encouraging effective and consistent condom use among males. Our results suggest that males’ knowledge about HIV/AIDS and its health consequences may be more likely to protect themselves from contracting or transmitting the virus, which might translate to engaging in less risky sexual activities through consistent condom use.

Our study showed that consistent condom use varied across socioeconomic strata. Males with primary, secondary, and higher levels of education, those in the middle, richer, and richest wealth index, and those from ethnic groups other than Hausa had higher odds of consistent condom use than their counterparts, a similar finding in previous studies [12,25,31]. A plausible explanation of our result could be that males with formal education may be exposed to health educational resources or high literacy levels to comprehend the risks associated with HIV/AIDS. Increased HIV-related knowledge among males with formal education might make them less receptive to misconceptions about HIV/STIs, understand its mode of transmission, or the repercussions on their overall health and quality of life. More so, having a formal education increases positive attitudes and behaviours by engaging in safe sex practices and consistent condom use. In addition, formal education could enable males to make informed decisions towards accepting condoms as effective family planning services than their counterparts without formal education. Our findings concerning wealth status indicate that increased wealth status removes financial barriers associated with accessing and using condoms consistently than their colleagues in the poor wealth index. Although in Nigeria, there are national strategies and efforts by civil organizations that aim to reduce condom inequalities [32,33], this study shines the light that gaps still exist. Moreover, considering that over 40% or 83 million Nigerians currently live below the country’s poverty line of $381.75 per year, and the clear linkages between poverty and literacy, this finding calls for urgent coordinated and targeted efforts to encourage consistent condom use, particularly among males with no formal education and low wealth status [34–36].

Concerning ethnicity, this current study found that being a Yoruba and Igbo male was associated with the likelihood of consistent condom use than those from the Hausa ethnic group. This finding coincides with the literature suggesting that Hausas predominantly practice Islam religion, which has been found to reduce the odds of family planning services [37,38]. Interestingly, our study also supports this position in that we found that males who were affiliated with Islam religion had a lower likelihood of consistent condom use. Previous studies in sub-Saharan Africa suggest that religious ideology is strongly associated with the acceptance of modern contraceptives. However, scholars assert that while religion may explain contraceptive use, other factors such as socioeconomic and demographic characteristics moderate religious belief that should be considered [39]. Our findings support this notion because, in Nigeria, Hausas and/or Islams are predominantly in the north, and the evidence shows that Northern Nigeria measures poorly across several health and economic indicators [40,41], which may very well explain our results. Accordingly, our findings call for strategies that localise HIV-related messages and contraceptive awareness campaigns, including condom use by engaging with religious leaders and mediums that appeal to religious beliefs among this population.

This current study also found that males who were currently married, cohabiting, or previously married had significantly lower odds of consistent condom use. This finding is expected and corroborates with previous studies suggesting that married people may be more actively trying to conceive, may have fewer partners, or engage in less risky sexual behaviours, thus are less likely to consistently use condoms than those who are single [42,43]. Although this result seems promising, there are noteworthy concerns. For example, researchers in Kenya found that males use condoms consistently with commercial sex workers but were less likely to consistently use condoms with their regular and casual sexual partners [22,44]. In addition, because MSMs contribute to a large share of HIV prevalence in Nigeria and some live a double life by getting married to or in sexual relationships with females, one could infer that males, regardless of marital status and/or gender orientation, may have inconsistent condom use. Moreover, Nigeria currently has a substantial-high birth rate, unintended pregnancy, and a population surge [45–47], highlighting the importance of creating strategies that adequately synthesize males both married and unmarried about modern contraceptives, including condoms.

### Limitations and strengths

Although this study significantly contributes to and extends the literature on consistent condom use in Nigeria, some inherent limitations must be considered. First, this study uses a cross-sectional design and, as such, may be prone to recall bias. In addition, establishing causality between variables may wane. Second, the key independent variable—HIV knowledge is broad and does not accurately measure what constitutes knowledge. As such, we focus on knowledge related to HIV transmission but are unable to establish the accuracy of the HIV knowledge reported. That is, if the knowledge is scientific or not. Based on this, we recommend that future studies measure other constructs related to HIV knowledge to effectively target and encourage condom use. Despite these limitations, this study utilises the most recent DHS data and provides recent estimates that could be generalisable to today’s clime. In addition, the current sample size infers a high statistical power, which improves generalisability beyond Nigeria.

## Conclusion and implications

After controlling for covariates, this current study established a strong association between HIV- transmission knowledge and consistent condom use among sexually active males in Nigeria. We also found that educational level, wealth index, and ethnicity are associated with condom use among this population, and therefore there is a need to look at the social determinants of sexual health. Findings from this current study call for targeted public health strategies at all government levels that integrate cultural and localized health education to combat the HIV/AIDS epidemic in Nigeria. The study emphasizes the impetus of providing more information on accessing family planning services in Nigeria to males with no knowledge of HIV transmission, those with no education, men cohabiting, those practicing Islam, men from Hausa ethnicity, and those residing in the Southern part of Nigeria. These categories should be included in any interventions directed towards accelerating family planning service use in Nigeria.

## Abbreviations

UNAIDS: Joint United Nations Programme on HIV/AIDS
NDHS: Nigerian Demographic and Health Surveys
UNICEF: United Nations International Children’s Emergency Fund
NACA: National Agency for the Control of AIDS
PEPFAR: President’s Emergency Plan for AIDS Relief
MSM: Men who have sex with men
USAID: United States Agency for International Development
STD: sexually transmitted diseases
CDC: Centers for disease control and prevention
LMICs: low-and-middle-income countries
NPC: National Population Commission
PCA: Principal Component Analysis
cOR: unadjusted odds ratio
AOR: adjusted odds ratios
CIs: confidence intervals
